# Temporal Structure in Cooperative Interactions: What Does the Timing of Exploitation Tell Us about Its Cost?

**DOI:** 10.1371/journal.pbio.1002371

**Published:** 2016-02-03

**Authors:** Jessica L. Barker, Judith L. Bronstein

**Affiliations:** Department of Ecology and Evolutionary Biology, University of Arizona, Tucson, Arizona, United States of America; University of Lausanne, SWITZERLAND

## Abstract

Exploitation in cooperative interactions both within and between species is widespread. Although it is assumed to be costly to be exploited, mechanisms to control exploitation are surprisingly rare, making the persistence of cooperation a fundamental paradox in evolutionary biology and ecology. Focusing on between-species cooperation (mutualism), we hypothesize that the temporal sequence in which exploitation occurs relative to cooperation affects its net costs and argue that this can help explain when and where control mechanisms are observed in nature. Our principal prediction is that when exploitation occurs late relative to cooperation, there should be little selection to limit its effects (analogous to “tolerated theft” in human cooperative groups). Although we focus on cases in which mutualists and exploiters are different individuals (of the same or different species), our inferences can readily be extended to cases in which individuals exhibit mixed cooperative-exploitative strategies. We demonstrate that temporal structure should be considered alongside spatial structure as an important process affecting the evolution of cooperation. We also provide testable predictions to guide future empirical research on interspecific as well as intraspecific cooperation.

## Abstract 2

Considering the timing of exploitation relative to cooperation can provide insight into two debated features of mutualism: the costs of being exploited and the resulting mechanisms to control exploitation.

## Introduction

**Fig 1:**
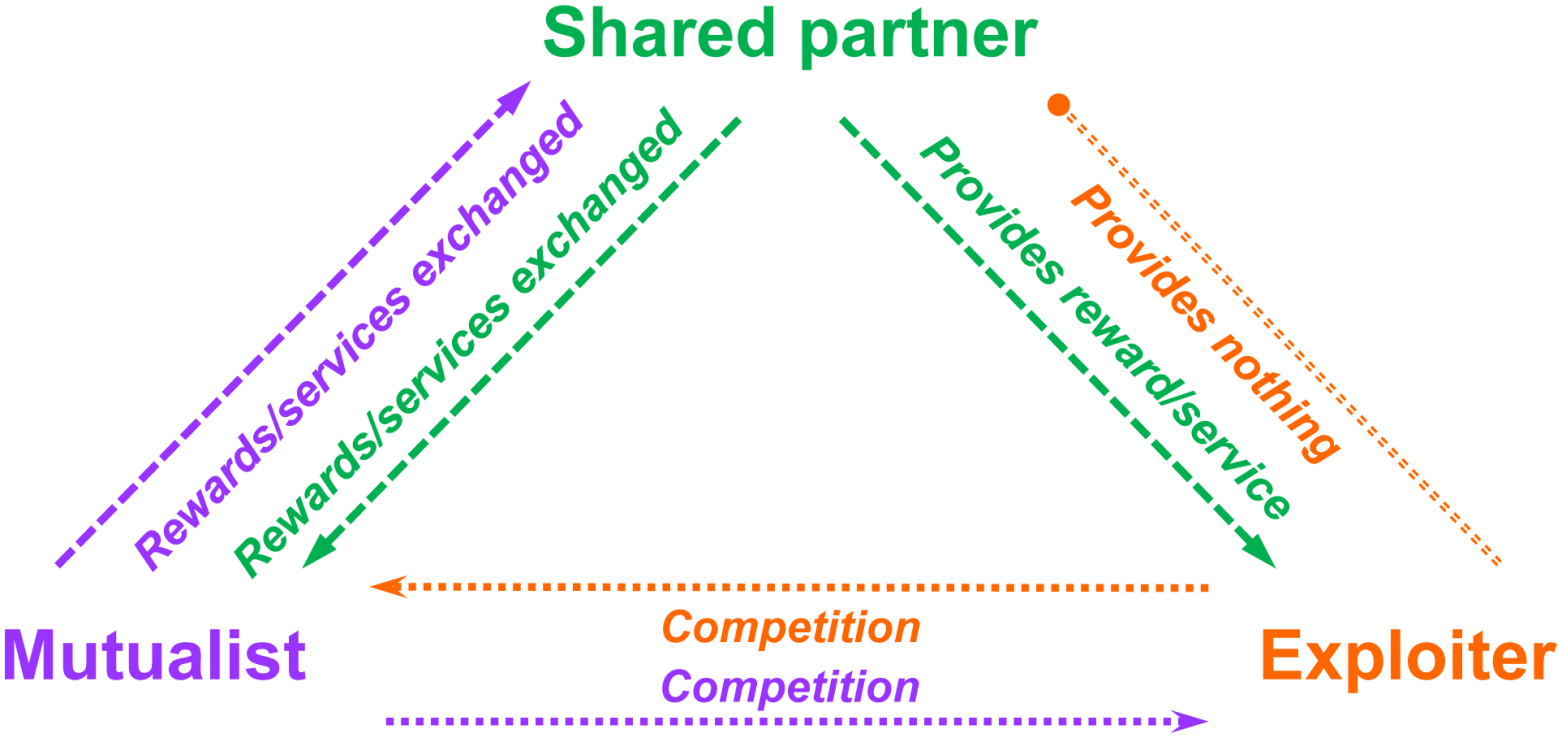
The three parties in the interactions we consider. The exploiter provides no reward or service in exchange for the commodity it takes from the shared partner and competes with the mutualist to obtain this commodity. We focus on cases where the mutualist and exploiter are different individuals exhibiting pure behavioral strategies, rather than a single individual that switches roles (a mixed strategy).

Cooperative interactions between species (hereafter, mutualisms) are ubiquitous, occurring among taxa from bacteria to animals [1–4]. In these interactions, heterospecific partners exchange commodities (rewards or services) that serve a variety of functions, including transport, protection, and nutrition [5]. However, there is also the potential for mutualists to be exploited by individuals that take commodities without providing any in return (Fig 1). It is clear that such behaviors, performed by purely exploitative individuals of the same or different species as well as by individuals that switch between mutualism and exploitation, are widespread in nature [6–9].

A major question is what determines the balance between cooperation and exploitation in mutualism [3,10,11]. Definitions of these behaviors have been discussed extensively elsewhere [6,11–14]. Here, we use “cooperation” to refer to any interaction in which an actor provides a benefit to a recipient, as this is the core concept common to the many definitions of cooperation, both within and among species [14]. The term “exploiter” is often used for any individual that takes but does not provide a benefit, including (a) individuals that can switch roles to become mutualists as well as those that cannot and (b) individuals evolutionarily derived from mutualists (including conspecifics) as well as those from separate evolutionary lineages (sometimes termed “parasites”). We focus the present analysis and examples on exploiters that cannot switch to be mutualists. Although the different identities and strategies of exploiters are critical from the perspective of how and when exploitation evolves, this issue largely falls outside the scope of the present inquiry, which focuses on the effects on and responses of the exploited individual.

Several hypotheses explain how mutualisms can persist despite the potential benefits of exploitation. For example, cooperation is maintained when partners’ fitness interests are aligned and when individuals can selectively associate with the most cooperative partners [15,16]. However, disagreements exist over the cost of being exploited and when mechanisms to prevent exploitation, such as sanctions or punishment, can be expected to evolve [10,17,18]. Here, we argue that the temporal sequence in which cooperation and exploitation take place can affect the cost of being exploited, and can thus help to explain when mechanisms to prevent exploitation will arise.

**Fig 2:**
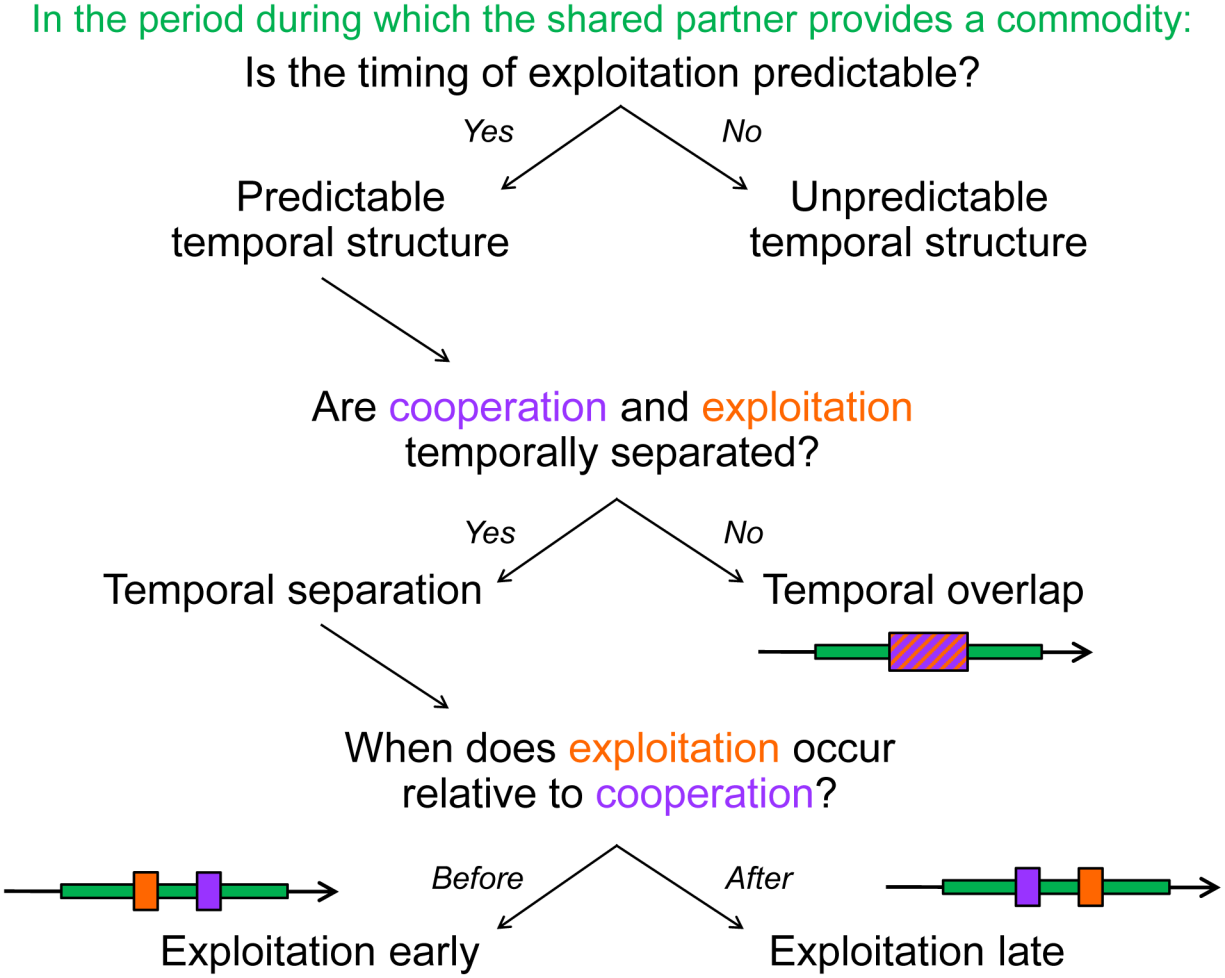
Categorizing temporal sequences of exploitation. The time period over which the shared partner provides a commodity is shown in green, and the times at which the mutualist and exploiter interact with the shared partner are shown in purple and orange, respectively. In this essay, we consider interactions with predictable temporal structure and ask how temporal overlap versus temporal separation (early versus late exploitation) affect the costs of and responses to being exploited.

In order to classify behaviors as cooperative or exploitative, the costs and benefits to both actors and recipients must be quantified [12,14,19]. In many cases, a given action by one party may have a different outcome (positive or negative) for another party depending on the context in which it occurs [20–22]. For example, it may be costly for a plant to host mutualistic ant defenders when the plant is not under attack by herbivores [18]. The context of an interaction often changes over time, and temporal variation in cooperation and exploitation from a single season to several years has been documented within mutualisms [23–25]. Here, we address a much shorter time scale: the time over which one individual provides a reward or service that can be used by another (Fig 2). Specifically, we consider the order in which actions take place within this time period. There is increasing awareness that the timing of interactions relative to each other has potentially important effects on the outcome of interactions between heterospecific as well as conspecific partners. For example, the timing of interactions influences queen–worker conflict in social insects [26], reproductive skew in communally breeding animals [27], selection of leaders by groups of migrating animals [28], and host–parasite interactions [29]. However, we lack explicit predictions about the ecological and evolutionary consequences of such “time-ordered” behaviors in either interspecific or intraspecific interactions [30,31].

In this essay, we focus on the order of exploitation relative to cooperation, specifically over the time period in which a mutualist provides a commodity (hereafter, the temporal sequence of cooperation and exploitation). This time period may be very different for different types of mutualism (e.g., a period of minutes in a cleaning mutualism versus a period of days in a pollinating seed-parasite mutualism; see details in Box 1 for pollinating seed-parasites). Here we focus on the ordering rather than on the duration of interactions. We first describe the different temporal sequences possible in mutualisms (Fig 2), using pollination examples to highlight phenomena whose natural history is well studied but whose temporal structure has not been explicitly documented (Box 1). We then develop predictions about how the temporal sequence affects the costs of and responses to being exploited, and suggest why different temporal sequences may evolve in different systems. In this analysis, we explicitly draw from concepts in behavioral ecology and social evolution (Box 2) and apply them to interspecific interactions.

**Box 1. Pollination Mutualisms: A Diversity of Temporal Sequences**

Plant-pollinator interactions are the most thoroughly studied mutualisms [32]. Plants provide a resource (usually nectar) to attract animals that carry out a service (transport of pollen). However, both plants and animals may exploit their partner by taking the proffered commodity without providing any in return. Certain plant species produce unrewarding flowers that attract visitors through mimicry [33]. Many floral visitors (“nectar robbers”) always obtain nectar by chewing through a flower’s corolla or by using the entryway made by others, in both cases bypassing pollen and stigmas [34]. Temporally separated exploitation is particularly well documented in pollinating seed-parasite (or nursery) mutualisms, a specialized form of pollination in which pollinators lay eggs (oviposit) in flowers and their offspring subsequently develop at the cost of a subset of the seeds [35]. These mutualisms are exploited by species that oviposit but never pollinate. Below we provide examples of each type of temporal sequence described in the main text (Fig 2).

**Temporal overlap**

Many nectarless orchids experience higher reproductive success when flowering simultaneously with rewarding species [36–38]. In some cases (particularly when unrewarding species mimic rewarding species [39]), this is because pollinators are attracted by the rewarding flowers and then remain to feed at the unrewarding ones (the magnet species hypothesis [40]). Temporal overlap can also result from phenological constraints: for example, both rewarding and unrewarding flowers may be constrained to bloom during a short period of pollinator abundance [41,42].

**Temporal separation (exploitation either early or late)**

In some cases, unrewarding flowers receive more visits if they are spatially or temporally separated from rewarding flowers [43–46]: pollinators may be less choosy when fewer resources are available (the remote habitat hypothesis [47]).

**Exploitation early**

Many nectarless orchids flower earlier than the rewarding species that they mimic [48,49]. This allows them to exploit naïve floral visitors before those visitors can learn to discriminate rewarding flowers [50]. Flowering early may be particularly important if each pollinator only makes a few visits or if visits to deceptive flowers are costly to the visitor [51]. Some species that exploit the fig–fig wasp pollinating seed-parasite mutualism can survive and reproduce in the inflorescences regardless of whether pollinators are present; they tend to locate inflorescences and lay their eggs before the pollinators arrive [52].

**Exploitation late**

Some nectar robbers are unable to chew holes themselves but can secondarily rob flowers through holes chewed by primary robbers [34]. This constrains secondary robbers to visit flowers later than primary robbers. Other exploiter species, however, require the pollinating actions of the mutualists to occur if they are to mature successfully: they feed on seeds and/or are unable to disrupt abscission of unpollinated inflorescences. These exploiters therefore delay their arrival until pollinators have visited [52–54].

**Box 2. Key Concepts from Social Evolution Theory**

Studies of intraspecific cooperative interactions, particularly in social groups, have yielded a large body of theory that predicts when conflict should arise over acquiring more resources for oneself versus cooperatively providing them to others, and who the recipients of cooperative resource provision should be. Some of these models of cooperation and conflict have been widely applied to interspecific mutualism, such as the Prisoner’s Dilemma [55,56], while the importance of many other concepts for understanding mutualism has been overlooked [57]. Here we highlight three concepts from social evolution that provide a rationale for the predictions we make about temporal sequences in mutualism, and that stress underlying similarities between intraspecific and interspecific cooperation.

**Diminishing returns and tolerated theft**

**Prediction about temporal sequences in mutualism:** Once benefits have already been acquired from a mutualist, the cost of subsequently being exploited is likely to be low (except for direct reproductive costs).

**Insight from social evolution:** Many social groups are characterized by diminishing returns of investing in cooperation or in conflict or exploitation [58]. A large investment in cooperation may produce disproportionately little benefit if only a threshold number of individuals is needed to produce a shared resource. An example is when a group of migrating animals requires a single leader [28]: as the leader pays an extra cost but gains no additional benefit, this scenario is the “volunteer’s dilemma” [59,60]. Similarly, a large investment in conflict (that is, obtaining more resources for oneself at the expense of others) may be disproportionately costly (e.g., lethal fighting) compared to a lower investment; this increasing cost of conflict can maintain cooperation in the face of the tragedy of the commons [58]. For example, food sharing in some human societies may have evolved as “tolerated theft:” if, after a big kill, a food owner has more meat than can be used before it spoils, then the cost of defending this food will outweigh the cost of letting others take it [61,62]. That is, the marginal value of food diminishes with the amount of food [63,64].

**Signal detection and kin recognition**

**Prediction about temporal sequences in mutualism:** It may be more difficult for the shared partner to discriminate among mutualists and exploiters when they overlap in time, because time cannot be used as a cue to distinguish partner identity [65].

**Insight from social evolution:** In the theory of animal communication, signals and cues provide information about a properties of the individual providing the signal or cue [66], and the receiver of the signal must distinguish an informative signal or cue from background noise, or between multiple classes of signal or cue, such as kin versus non-kin [67]. Signal detection theory quantifies the tradeoff between incorrectly responding to an absent signal and incorrectly ignoring a signal that is present; receivers are selected to minimize both false positives and false negatives [68]. When there is greater overlap between different classes of signal or cue (for example, when brood parasitic eggs are morphologically similar to the mother’s eggs [69]), it is more difficult to distinguish between them, as there is greater potential for costly error.

## Characterizing Temporal Sequences of Exploitation in Mutualism

We consider three parties (Fig 1): (1) the mutualist, which takes a commodity (a reward or service) from and provides a commodity to the shared partner; (2) the shared partner, which similarly takes a commodity from and provides a commodity to the mutualist; and (3) the exploiter, which takes a commodity from the shared partner but (unlike the mutualist) provides nothing in return. The exchange between the shared partner and the mutualist is referred to as cooperation, and the interaction between the shared partner and the exploiter is referred to as exploitation. We focus here on cases in which the exploiter and mutualist are different individuals (from the same or different species), rather than the same individual exhibiting different behaviors. It is likely that the exploiter and the mutualist are competitors, as they both use commodities offered by the shared partner (Fig 1). We define “temporal structure” as the sequence in which the exploiter and the mutualist interact with their common, shared partner while it is offering mutualistic commodities. We note the parallel with spatial structure, which is well documented as affecting the evolution of cooperation in diverse systems [70–72]: both spatial and temporal structure determine whether cooperators co-occur with other cooperators or with exploiters. Below and in Fig 2, we categorize these temporal associations by considering whether exploitation predictably overlaps with or is separated from cooperation. In Box 1, we use pollination mutualisms as illustrations of different temporally structured sequences.

### 1. Predictability of Temporal Structure

The sequence in which a mutualist and exploiter interact with the shared partner may be unpredictable. (Note that this does not preclude distinct temporal sequences of cooperation and exploitation; rather, which event occurs first may vary.) For the rest of this essay, we focus on temporally structured interactions in which sequences of cooperation and exploitation are predictable and examine the consequences of this predictable temporal structure on the cost of being exploited.

### 2. Temporal Overlap

If the timing of exploitation is predictable, one possibility is that exploitation and cooperation occur simultaneously. This is the case in many symbioses, in which hosts simultaneously harbor and interact with cooperative and uncooperative symbiont strains [9], as well as some ant protection mutualisms, in which multiple ant species differing in quality as mutualistic defenders simultaneously interact with a host plant [73,74].

### 3. Temporal Separation

In other temporally structured interactions, the exploiter and mutualist predictably interact with the shared partner at distinct times. For example, ant species that differ in protective ability may have distinct thermal niches, such that a plant interacts with only one of them at a given time [75,76]. We define these interactions as temporally separated. The exploiter may always interact with the shared partner before the mutualist does, a pattern we term “exploitation early;” as we discuss in more detail below, this may happen if exploiters deter mutualists, as in some ant-protection mutualisms [77,78] and seed-dispersal mutualisms [79]. In other cases, the exploiter may always interact with the shared partner after the mutualist does, which we term “exploitation late.” For example, some animals pilfer and consume seeds hidden by mutualistic, seed-caching granivores [80].

## How Does the Temporal Sequence Affect the Net Costs of Being Exploited?

In order to understand the consequences of temporally structured cooperation and exploitation, we adopt the perspective of the shared partner and predict how the net costs of interacting with an exploiter are mediated by its timing relative to the interaction with a mutualist (Fig 2). To assess the net costs to the shared partner, we consider both the costs incurred by interacting with the exploiter and the benefits accrued by interacting with the mutualist (summarized in Table 1).

**Table 1:** Summary of predictions about temporal sequences of exploitation. For each temporal sequence, we predict how the timing of interactions affects the net cost to the shared partner and potential responses by the shared partner as a result of this cost. We also predict selection pressures on the mutualist and exploiter that may cause each type of temporal sequence to arise.

Temporal Sequence	Net Cost to Shared Partner of Being Exploited^1^	Shared Partner’s Responses to Being Exploited	Factors Affecting Mutualist’s and Exploiter’s Timing^2^
Temporal overlap	High: shared partner may not yet have acquired benefits from mutualist	Trade-off with deterring mutualists, and discrimination difficult: if there is a response, expect tolerance rather than resistance; if resistance, expect directed deterrence	Exploiters evade detection by “hiding” among mutualists
Temporal separation: exploitation early	High: shared partner may not yet have acquired benefits from mutualist; future mutualists may be deterred by prior actions of exploiters	Trade-off with deterring mutualists selects for tolerance rather than resistance; if resistance, expect directed deterrence	Exploiters evade detection by interacting with naïve shared partner
			Competition among mutualists and exploiters; exploiters are superior competitors
Temporal separation: exploitation late	Low: shared partner has already acquired commodity from mutualist; “tolerated theft” of leftover rewards	Little benefit of deterring exploiters: thus, weak selection to respond	Exploitation dependent on prior actions of mutualists
			Competition among mutualists and exploiters; mutualists are superior competitors

^1^If the shared partner incurs direct reproductive costs, the net cost of being exploited will be high regardless of the temporal sequence. This in turn will likely select for responses to avoid being exploited.

^2^All temporal sequences may be affected by external factors such as species-specific life histories and environmental conditions such as temperature.

If the shared partner has already received a reward or service from a mutualist, then there may be little or no additional benefit to acquiring more [54,81,82]; that is, there may be diminishing returns over time, as described in Box 2 [58]. In addition, if rewards are relatively cheap for the shared partner to produce, such that some are left over after interaction with mutualists, then relinquishing these rewards to late exploiters is not costly, e.g., if residual nectar is robbed after a flower has already been fully pollinated and its pollen fully dispersed [83]. This taking of excess rewards is analogous to food sharing in humans as a form of “tolerated theft” [61–64], defined in Box 2, in which the cost of others taking from a surplus of food is low. Thus, we predict that costs incurred by the shared partner from the exploiter using up resources will, all else equal, be lower when exploitation happens late than when exploitation is early or in temporal overlap with cooperation.

The shared partner may incur direct costs from exploitation, such as damage to reproductive tissue itself or somatic damage that precludes future reproduction. Alternatively, the costs may be indirect, such as experiencing reduced encounters with mutualists (an opportunity cost) or having to produce more rewards in order to attract mutualists. If exploitation imposes direct damage to reproductive tissue, we predict that its cost will be high regardless of the temporal sequence of the interaction. However, the magnitude of opportunity costs depends on the shared partner’s need for and probability of future interactions with mutualists. We predict that if exploitation happens early, or overlaps in time with cooperation, indirect costs will be higher than if exploitation takes place later, as subsequent mutualists will be deterred.

## As a Result of These Costs, What Should the Shared Partner Do?

Given that we expect the costs of being exploited to depend in part on the timing of exploitation relative to cooperation, the magnitude and type of the shared partner’s response to being exploited should also depend on the temporal sequence of exploitation. Here, we predict whether and how the shared partner will respond (summarized in Table 1). We expect no response if being exploited is not costly, if the cost of a response exceeds its benefits, or if there are constraints that prevent an effective response. In cases in which the shared partner does respond, potential responses include resistance to exploiters, in which the shared partner minimizes the frequency of interactions with them, and tolerance to exploiters, in which the shared partner instead minimizes the costs of those interactions [84,85]. Below we outline when each of these responses is expected to arise, based on the net costs of being exploited in different temporal sequences.

### Tolerance Arising from Inability to Detect Exploiters

We predicted above that exploiters could evade detection if they co-occur with mutualists. One mechanism is that temporal separation can be a cue used by the shared partner to distinguish exploiters from mutualists: for example, if cooperation generally precedes exploitation, then late arrival is a cue for identifying exploiters. The mechanisms and consequences of distinguishing between two classes of potential partner have been addressed in the intraspecific cooperation literature by signal detection theory (Box 2). The well-studied example of how birds distinguish between brood parasitic eggs and host eggs based on morphological similarity [69] provides insight into the case of how effectively mutualists can be distinguished from exploiters based on temporal overlap of interactions: the greater the overlap, the more errors shared partners can be expected to make. We predict that when distinguishing exploiters from mutualists is difficult or impossible (e.g., when there is temporal overlap), the shared partner is more likely to mitigate the cost of exploitation via tolerance of its effects rather than via resistance. For example, mass events of seed production (masting) may satiate seed predators while attracting seed dispersers [86–88]. Tolerance has also been suggested as a mechanism by which plants cope with the effects of nectar robbers [89].

### Directed Deterrence Arising When Temporally Overlapping Exploiters Can Be Detected

In some cases, the shared partner can distinguish between mutualists and exploiters even when their actions are simultaneous. In these cases, we predict that the shared partner may evolve resistance and that its defenses will be selective (“directed deterrence”). This may take the form of reward chemistry that deters exploiters but not mutualists [90,91] or morphological adaptations that allow only mutualists access to rewards [92,93]. In addition, the shared partner may be able to shift the temporal sequence of exploitation from overlap to separation: for example, matching the time of flowering or nectar production to the peak activity of pollinators but not to that of nectar robbers [23,94].

### Tolerance and Directed Deterrence Arising from Costs of Deterring Future Mutualists

When exploitation occurs early, the benefit of deterring exploiters will be high. We predict this will select for mechanisms to control early exploitation. For example, some ant-defended plants can abort domatia (modified leaves housing the ants) before nonmutualistic ants inflict direct reproductive damage by castrating the plant’s flowers [95]. However, there is a trade-off: when exploitation occurs early, the cost of deterring future mutualists is also high, and this may limit the evolution of the control of early exploitation. This trade-off may select for directed deterrence, as described above, as well as for mechanisms of tolerance to exploiters rather than resistance to them.

### No Response to Late Exploitation, Unless Commodities Are Costly to Produce

For any temporal sequence of exploitation, the shared partner is expected to optimize production of commodities [96,97]. If the commodities are costly to produce and cooperation predictably occurs before exploitation, there should be selection for the shared party to reduce the production of commodities such that there are none left to be taken by exploiters. However, if it is not costly for excess commodities to remain after the interaction with mutualists and if additional costs of being exploited late (e.g., reduced future interaction rate with mutualists) are generally low or nonexistent, we predict that the shared partner will not respond to late exploitation. For example, the low cost of residual nectar being removed after pollination has occurred may be one reason why plants rarely show adaptations to deter nectar robbers [34].

## Why Do the Mutualist and Exploiter Interact with the Shared Partner at Certain Times?

We have now demonstrated that a range of temporal sequences of exploitation exists in mutualism (exemplified by pollination mutualisms: Box 1) and that different temporal sequences select for the shared partner to respond in different ways to being exploited (Table 1). Yet, there remains the question of why different temporal sequences exist. Thus far, we have taken the perspective of the shared partner. However, the temporal sequences that we see in nature are due to selection not just on the shared partner but also on the mutualist and the exploiter. The combination of these separate selection pressures can yield a wealth of evolutionary trajectories constrained to some extent by system-specific natural history. A full treatment of this important question is beyond the purview of this essay. Here, we suggest how different outcomes may result from three key aspects of the natural history of exploitation (summarized in Table 1).

### Detection by Shared Partner

If the shared partner can detect an exploiter, it can in some cases choose not to affiliate with that exploiter or can terminate the interaction after being exploited. This is the case for the clients of the cleaner wrasse *Labroides dimidiatus* [98]. Thus, success of exploitation may depend on evading detection by the shared partner. Exploiters can evade detection if they “hide” among temporally overlapping mutualists, for example, when Batesian mimics co-occur with Müllerian mimics [99]. Exploiters can also evade detection if they interact with a naïve shared partner that has not yet interacted with mutualists, i.e., if exploitation occurs early.

### External Constraints

External factors may constrain mutualists and exploiters to interact with the shared partner at certain times. This constraint can result in predictable overlap or temporal separation in different cases. For example, temporal overlap may arise if mutualists and exploiters share the same food sources, while temporal separation may occur if mutualists and exploiters are most active at different temperatures. Opportunities for exploitation may also be dependent upon the prior actions of other individuals. This would be the case if, for example, the commodity taken by an exploiter is produced after the shared partner interacts with a mutualist, or is a product of the mutualism. For example, seed predators consume the product of pollination mutualisms.

### Competition between Mutualist and Exploiter

Mutualists and exploiters may avoid competition for commodities provided by the shared partner if they interact with the shared partner at different times and if commodities are either produced in excess or are not completely depleted during the interaction. However, if the quality or quantity of commodities decays over time, then selection should favor both the mutualist and exploiter taking the commodities first. For example, extrafloral nectar to attract ant defenders is often produced for only a limited time [100]). All else being equal, competition between mutualists and exploiters should result in early exploitation if the exploiter is the superior competitor and late exploitation if the mutualist is superior.

## Discussion

Different temporal sequences of cooperation and exploitation have been documented in many mutualisms (Box 1) [54,82], but the ecological and evolutionary significance of the timing of exploitation has not previously been addressed. Temporal structure, like spatial structure, can determine which classes of individuals (exploiters and mutualists) interact with each other, and thus the temporal sequence of exploitation is likely to affect the dynamics of cooperation in mutualism. We have proposed a framework to categorize the variety of temporal sequences (Fig 2), focusing on interactions in which the timing of exploitation and cooperation is predictable. The predictability of a temporal sequence may affect the shared partner’s potential responses to being exploited: for example, if the timing of exploitation is unpredictable, it may be more costly to deter potential partners than if exploitation is predictably later than cooperation. However, the predictions we make about the consequences for the shared partner in a given temporal sequence still hold regardless of whether that sequence is predictable. Future research should focus on identifying the factors that affect temporal predictability. For example, we would expect predictable temporal sequences if the mutualist and exploiter are constrained by different external factors, such as distinct thermal tolerances or different natural enemies.

We concentrated in this essay on interactions in which exploiters and mutualists are different individuals. These include cases in which exploiters are either distinct species or individuals within species polymorphic for cooperation and exploitation. However, in other systems, individuals can choose to either cooperate or exploit their partners [6]. The cost of being exploited within a given temporal sequence should not be affected by the strategy set of the exploiter (i.e., whether it can switch to mutualistic behavior or not). However, these different classes of exploitation are increasingly recognized to have different evolutionary origins and ecological dynamics [3,6,11,12] and may affect the shared partner’s responses. For example, it may be more difficult to deter exploiters without also deterring mutualists when exploiters and mutualists are the same individuals.

Further studies should also explore the conditions under which specific temporal sequences arise. The different classes of exploitation described above may exhibit different temporal structures. For example, when mutualists and exploiters are different individuals, they will potentially be competing with each other, whereas an individual switching between mutualism and exploitation cannot be competing with itself. It is also important to determine whether certain temporal sequences are more commonly associated with (1) certain behavioral options available to each party, e.g., whether an individual has outside options for obtaining resources [53]; (2) different classes of commodities, i.e., transportation, nutrition and protection [53]; and (3) interactions whose outcome depends upon the presence of a third party, e.g., ant protection mutualisms [20].

A tractable and promising set of future inquiries emerge from the predictions we have made about how the timing of exploitation affects the cost of and response to being exploited, taking the perspective of the shared partner (Table 1). To develop these predictions, we have drawn on theory from intraspecific cooperation (Box 2). Helpful parallels with temporal sequences in mutualism can be found in the literature on tolerated theft, in which sharing food is not costly if there is surplus [61–64], and kin recognition, in which the ability to discriminate between two parties depends on their overlap [67]. Cooperation among conspecifics in social groups has been studied primarily from a behavioral perspective that overlaps relatively little with the community and population ecology approach applied to mutualism. Although intra- and interspecific cooperation are in many ways similar, few concepts developed in one field have yet been applied to the other, despite the potential for new cross-disciplinary insights [101].

The concept of temporal sequences of cooperation and exploitation is in turn not limited to mutualism: this framework applies to any interaction in which benefits are exchanged, including cooperation within species. As an example from intraspecific social groups, chacma baboon (*Papio ursinus*) subordinates groom dominants in exchange for access to feeding sites and exhibit early cooperation as a result of competition for these foraging sites [102]. The temporal structure of exploitation in mutualism also has intriguing parallels with the dynamics of virulence in host–parasite interactions. For example, the outcome for a host infected by multiple genetically distinct malaria strains depended on whether the host was inoculated simultaneously or sequentially [103]. As another example, high virulence late in the course of an infection is less likely to impact transmission, just as we predicted here that exploitation late in mutualism is generally less costly [104]. There is also evidence that exploitation occurs late in long-lived populations of microbes and that regulatory mechanisms operate to reduce costly early exploitation [105]. This system also points towards the importance of suppressing competition in maintaining cooperation [106–108].

The predictions we make here could be tested by quantifying the costs and benefits accrued by each party in interactions with different temporal sequences. This approach would yield the net fitness effects on each party and reveal the evolutionary consequences of temporal sequences of exploitation in different mutualisms and intraspecific systems. As an example, experimental manipulations of the timing of exploitation could be implemented in studies of nectar robbing, e.g., by comparing fruit production by flowers visited by pollinators before and after visits by nectar robbers. Data from such experiments that use temporal sequences as a conceptual framework will help shed light on the unresolved issues of the occurrence of exploitation, the costs of being exploited, and the evolution of mechanisms to control exploitation.
